# Financial distress among breast cancer survivors

**DOI:** 10.25082/CCR.2020.01.004

**Published:** 2020-08-03

**Authors:** Steven S. Coughlin, Deepak Nag Ayyala, Martha S. Tingen, Jorge E. Cortes

**Affiliations:** 1 Department of Population Health Sciences, Medical College of Georgia, Augusta University, Augusta, GA; 2 Institute of Public and Preventive Health, Augusta University, Augusta, GA; 3 Department of Medicine, Medical College of Georgia, Augusta University, Augusta, GA; 4 Georgia Cancer Center, Augusta University, Augusta, GA

**Keywords:** breast cancer survivors, costs, financial distress

## Abstract

**Aims:**

there has been an increasing awareness of the potential for oncology care to result in long-term financial burdens and financial toxicity. Patients who report cancer-related financial problems or high costs are more likely to forgo or delay prescription medications and medical care.

**Materials and Methods:**

we examined financial distress using data from a survey of 164 breast cancer survivors who had completed primary therapy for the disease.

**Key Findings:**

among respondents, 8.6% (13 of 151) reported that “being less able to provide for the financial needs of their family” was as a severe problem; 14.4% (22 of 153) reported “difficulty in meeting medical expenses” was a severe problem; and 8.4% (13 of 154) reported that “no money for cost of or co-payment for medical visits” was a severe problem. About 8.4% (13 of 154) of the respondents reported that “no money for cost of or co-payment for medicine” was a severe problem. In logistic regression analysis, younger age and lower household income were significant predictors of financial distress. In multiple linear regression analysis, younger age and lower household income were significant predictors of financial distress.

**Significance:**

financial toxicity remains a major issue in breast cancer care. Efforts are needed to ensure patients experiencing high levels of financial toxicity are able to access recommended care. In addition, patients should talk with their providers about the costs of oncology care and about opportunities to reduce costs while maintaining high quality of care.

## 1 Introduction

There has been increasing awareness of the potential for oncology care to result in long-term financial burdens and financial toxicity to patients and their families.^1–3^ About 28% to 48% of cancer survivors experience financial toxicity based upon monetary measures and 16% to 73% experience financial toxicity based upon subjective measures.^4^ Sources of financial distress include costs associated with cancer care services (*e.g.*, medications, supplies, co-payments, transportation, parking) and reduced income because of missing work, loss of employment, or unplanned retirement.^5^ Cancer survivors may be vulnerable to out-of-pocket expenses due to unemployment, medical debt, and diminished consumer credit.^6,7^ This is especially true for women, young patients, racial and ethnic minorities, persons who have low income or financial illiteracy, and those without health insurance.^1,4,8^ Cancer survivors with public insurance experience greater economic burden than those with private insurance,^9^ although having health insurance does not fully protect against financial distress associated with cancer.^8^ Among low-income women, the costs of breast cancer care has been reported to represent up to 98% of an individual’s annual earnings.^3,10^ High cancer-related financial burden affects treatment choice, treatment compliance, and cancer outcomes.^8^ Patients who report cancer-related financial problems or high costs may be more likely to forgo or delay prescription medications or medical care^4,11^ Financial distress due to financial obligations, debt and diminished wealth may interfere with the ability of cancer patients to cope with physical symptoms and follow-up care, and lead to poorer health-related quality of life and health outcomes.^4^

We examined financial distress using data from a survey of 164 breast cancer survivors who had completed primary therapy for the disease. The overall objective was to determine the prevalence of financial strain and financial distress, and to identify predictors of financial distress.

## 2 Methods

The Cardiovascular Disease outcomes among Breast Cancer Survivors Study (CVDBCS) was a postal survey of a multiethnic cohort of breast cancer survivors who reside in Augusta, GA and who had been treated at Augusta University Health or the Georgia Cancer Center. Non-institutionalized women were eligible to take part in the study if they resided in Augusta-Richmond County and Columbia County, GA, or Aiken County, SC and had been diagnosed with stage I-IV breast cancer and completed primary therapy for the disease (chemotherapy, radiation, surgery).

Data were collected using postal survey questionnaires and via abstraction of electronic medical records. The mailings were sent to 1,000 randomly sampled potential research participants who had been treated at Augusta University Health or the Georgia Cancer Center after July 2019. A sequential mailing protocol was followed using a modified Dillman method. An advance letter was mailed to the women by the study principal investigator. The letter provided information about the study (purpose, potential benefits, and risks) and informed them that they could opt out and not receive further mailings about the study. Three weeks later, an informed consent letter was mailed to women who had not opted out along with a copy of the questionnaire and a pre-addressed, stamped return envelope. Women who had not opted out or returned a completed questionnaire were sent a reminder postcard four weeks later. Survey responses were checked for completeness and then coded and entered into an electronic database. Survey questions about breast cancer diagnosis were obtained from a previous study of breast cancer survivors.^12^ Respondents were asked about a variety of symptoms and problems in living: “Tell us about your experience as a survivor. Below is a list of problems that people may have after cancer treatment. Please check the box to show how much this has been a problem for you during the past month (Not a problem, Somewhat a problem, A severe problem).” The inventory of symptoms and challenges in living included “Being less able to provide for the financial needs of my family’^1^, “Difficulty in meeting my medical expenses”, “No money for cost of or co-payment for medical visits”, and “No money for cost or co-payment for medicine”.

After crosstabulations and exploratory analyses of the survey data were completed, logistic regression methods were used to compare groups of breast cancer survivors who did or did not report financial strain or financial distress according to age, race, education, household income, stage-at-diagnosis, years since breast cancer diagnosis, cancer treatment, hypercholesterolemia, hypertension, diabetes, congestive heart failure, and smoking status. The dependent variable in these analyses was whether or not the respondent answered at least one of the four questions about financial distress as somewhat/severe distress.

Potential confounding factors were controlled for in these analyses. Ninety-five percent confidence intervals were obtained for adjusted odds ratios. Levels of statistical significance were determined using Wald chi-square tests and Log-likelihood ratio tests. The goodness-of-fit of each model was examined using the Log-likelihood ratio tests.

Following the logistic regression analysis, multiple linear regression techniques were used to examine predictors of financial strain or distress according to age, race, education, household income, stage-at diagnosis, years since breast cancer diagnosis, cancer treatment, hypercholesterolemia, hypertension, diabetes, congestive heart failure, and smoking status. The dependent variable in these analyses was a quantitative score defined as follows: No stress was scored as zero, somewhat/severe distress was scored as 1, and the total distress was defined as the total score over the four questions.

## 3 Results

A total of 164 women completed the study questions (response rate 16.4%). The mean age of the women was 67 years (SD: 41.1) (Table 1). Among all participants, 66.7% were white, 29.5% were African-American, and the remainder were of other races. More than half (58.4%) of the women were insured through Medicare and 29.2% held private insurance. With respect to breast cancer stage at diagnosis, 19.8% of the women had ductal carcinoma in situ, 26.8% had stage I disease, 21.0% had stage II disease, 8.9% had stage III disease, and 5.1% had stage IV disease. The mean number of years since diagnosis was 9.4 years (SD: 8.8). About 54.9% of the women reported receiving chemotherapy; 45.1% reported receiving hormonal therapy; and only 4.9% reported biologic/targeted therapy.

About 8.6% (13 of 151) of the respondents reported that “being less able to provide for the financial needs of their family” was a severe problem (Table 2). Nearly 14.4% (22 of 153) of the respondents reported “difficulty in meeting medical expenses” was a severe problem (Table 2). Approximately 8.4% (13 of 154) of the respondents reported that “no money for cost of or co-payment for medical visits” was a severe problem (Table 2). About 8.4% (13 of 154) of the respondents reported that “no money for cost of or co-payment for medicine(s)” was a severe problem (Table 2). About 65.81% of the respondents (102 of 155) answered at least one of the four questions about financial distress as somewhat/severe problem (results not shown).

In logistic regression analysis (Table 3), younger age and lower household income were significant predictors of financial distress. Having an associate degree was of borderline significance. In multiple linear regression analysis (Table 4), younger age, and lower household income were significant predictors of financial distress.

## 4 Discussion

The results of this survey indicate that over 8% of the breast cancer survivors in this sample are having severe difficulty paying for medical visits or for the cost of medications. Over 8% reported that providing for the financial needs of their family was a severe problem. A total of 65.81% of the respondents (102 of 155) answered at least one of the four questions about financial distress as a somewhat/severe problem. Not being able to afford household expenses is one of the most commonly reported reasons for delayed medical care among cancer patients.^13^

In multivariate analysis with logistic regression or multivariate linear regression, younger age and lower household income were significant predictors of financial distress in the current study. Our results are consistent with previous studies that indicate that younger age and low income are associated with financial toxicity among cancer patients.^4,14,15^ Younger women are less likely to have Medicare insurance and they may also have expenses associated with childrearing.

The number of cancer treatment options has significantly increased over the past two decades, leading to improvement in patients outcomes for many malignancy types.^14^ These advancements, however, are costly and cancer is now the second most expensive disease in the United States, after heart disease.^14^ As the cost of oncology care escalates, financial concerns of patients, families, physicians, and health care systems are increasingly common.^15,16^

The causes of financial distress among cancer patients is multifactorial and includes patient demographics and socioeconomic characteristics, disease characteristics, treatment characteristics, and healthcare system factors.^14^ In the current study, younger patients and those with a low-income or less educational attainment were more likely to report financial distress. Because we lacked information about healthcare system factors,^14^ the current study does not provide any information about the contribution of healthcare system factors to financial distress among breast cancer survivors. We did not observe any statistically significant associations with disease characteristics (*e.g.*, stage at diagnosis) or type of treatment received.

As noted by Lentz *et al*.,^14^ health care providers can take several steps to reduce financial toxicity including: 1) consider cost in addition to adverse effects if multiple treatment regimens have similar efficacy; 2) provide patients with estimates of cancer care costs; 3) consider whether an intervention will provide meaningful improvement; 4) incorporate financial toxicity screening into clinic evaluation and workflow; and 5) educate and provide assistance to patients about insurance benefits, other financial aid that may be available to them, etc. Providers such as medical, surgical, and radiation oncologists have a critical role to play in addressing financial toxicity. Early referral to a financial navigator should be considered when a need is identified.^14^ A multidisciplinary approach involving nurses, social workers, and financial navigators provides additional expertise. Financial navigators can assess any risk of financial toxicity at time of cancer diagnosis and provide financial education and counseling.^14^

With respect to other limitations, misclassification bias is a possibility due to the use of self-reported information. The current study was cross-sectional in nature which precludes conclusions on financial impact over a period of time. An additional limitation is the results of this study may not be generalizable to other populations of breast cancer survivors. However, the sample was somewhat diverse by race, socioeconomic factors, and history of breast cancer diagnosis and treatment.

Financial toxicity remains a major issue in breast cancer care. A significant number of cancer patients and families struggle with financial difficulty.^15^ Financial distress may be seen as distinct from, but not isolated from, the overall anxiety and discomfort experienced as a result of the cancer diagnosis and resulting treatment(s).^4^ Financial distress from mounting financial obligations and debt may interfere with the patient’s ability to cope effectively with cancer and its treatment, thereby adversely affecting health outcomes.^4^ Identifying those patients most at risk of facing financial difficulty is an important measure to ensure safety nets are available for these targeted populations.^15^ Further studies are needed that include a larger percentage of minority patients as well as rural and underserved patients experiencing cancer and its resulting treatment(s). Efforts are needed to ensure all patients experiencing high levels of financial toxicity are able to access recommended care.

## Figures and Tables

**Table 1:** Characteristics of study participants (n=164)

Characteristic	Frequency (%)
**Age** (years) mean (SD) (N=163)	67 (41.1)
**Race** (N = 156)	
White, Non-Hispanic	104 (66.7)
African American, Non-Hispanic	46 (29.5)
Other^1^	6 (3.9)
**Annual Income** (N = 46)	
<$20,000	17 (10.4)
$20,000 - $34,999	17 (10.4)
$35,000 - $49,999	17 (10.4)
$50,000 - $64,999	14 (8.5)
$65,000 - $79,999	8 (4.9)
$80,000 +	38 (23.2)
Missing^2^	53 (32.3)
**Number of people in household** (N = 160)	
1	48 (30.0)
2	83 (51.9)
3 +	29 (18.1)
**Employment status** (N = 163)	
Retired	99 (60.7)
Employed	34 (20.9)
On disability	16 (9.8)
Homemaker	9 (5.5)
Temporarily unemployed	4 (2.5)
**Marital status** (N = 163)	
Married/Partner	84 (51.5)
Single	24 (14.7)
Widowed	32 (19.6)
Separated/Divorced	23 (14.1)
**Education** (N = 157)	
Less than HS	5 (3.2)
HS or equivalent	42 (26.8)
Some college	27 (17.2)
Associate degree	22 (14.0)
Bachelor degree	27 (17.2)
Graduate degree	34 (21.7)
**Health Insurance** (N = 161)	
Medicare	94 (58.4)
Private insurance	47 (29.2)
Other^3^	20 (12.4)
**Perceived general health** (N = 162)	
Excellent	16 (9.9)
Very good	58 (35.8)
Good	59 (36.4)
Fair	24 (14.8)
Poor	5 (3.1)
**Breast cancer stage at diagnosis** (N = 157)	
Ductal carcinoma in situ	31 (19.8)
Stage I	42 (26.8)
Stage II	33 (21.0)
Stage III	14 (8.9)
Stage IV	8 (5.1)
Don’t know	29 (18.5)
**Time since diagnosis (in years) mean** (SD) (N = 155)	9.4 (8.8)
**Type of treatment received**^4^ (N = 164)	
None	2 (1.2)
Surgery	161 (98.2)
Radiation	111 (67.7)
Chemotherapy	90 (54.9)
Hormone therapy	74 (45.1)
Biologic/Targeted therapy	8 (4.9)

**Table 2:** Self-reported history of financial distress among breast cancer survivors (n = 164)

Being less able to provide for the financial needs of my family	
Not a problem	116 (76.82%)
Somewhat a problem	22 (14.57%)
A severe problem	13 (8.61%)
Difficulty in meeting my medical expenses	
Not a problem	108 (70.59%)
Somewhat a problem	23 (15.03%)
A severe problem	22 (14.38%)
No money for cost of or co-payment for medical visits	
Not a problem	125 (81.17%)
Somewhat a problem	16 (10.39%)
A severe problem	13 (8.44%)
No money for cost of or co-payment for medicine	
Not a problem	128 (83.12%)
Somewhat a problem	13 (8.44%)
A severe problem	13 (8.44%)

**Table 3:** Predictors of financial distress among breast cancer survivors from logistic regression analysis (n = 164)

Covariate	Odds Ratio	OR Confidence Interval	p-value
Age	0.823	0.7127 – 0.9112	0.0013
Race - White	Referent		
Race - African American	0.9134	0.0877 – 9.699	0.9377
Race - Other	32.2569	0.4334 – 4497.288	0.1227
Stage - DCIS	Referent		
Stage I	0.3031	0.0107 – 6.435	0.4507
Stage II	0.5921	0.0161 – 20.0556	0.7659
Stage III	0.2622	0.0027 – 13.8554	0.5307
Stage IV	37.5132	0.3774 – 9668.54	0.1442
Unknown Stage	0.1153	0.0014 – 4.1836	0.2674
Education - Less than High School	Referent		
Education - Some college	2.8747	0.0796 – 141.7124	0.5657
Education - Associate Degree	29.3424	1.362 – 2580.691	0.0636
Education Bachelor’s Degree	0.1015	2e-04 – 10.651	0.3923
Education - Graduate Degree	1.2416	0.0175 – 80.3945	0.917
Income - < $20,000	Referent		
Income - $20,000 – $34,999	0.0081	0 – 0.4612	0.0361
Income - $35,000 – $49,999	9.00E-04	0 – 0.0564	0.005
Income - $50,000 – $64,999	1.94E-05	0 – 0.0045	0.0018
Income - $65,000 – $79,999	2.01E-05	0 – 0.0179	0.0133
Income - > $80,000	9.00E-04	0 – 0.0609	0.0063
Time since diagnosis	1.0305	0.9022 – 1.1899	0.6605
Treatment - Radiation	8.1986	0.6816 – 205.3786	0.1357
Treatment - Chemotherapy	2.5642	0.1885 – 48.1497	0.4916
Treatment - Hormone	0.7243	0.068 – 6.4841	0.7703
Treatment - Targeted	73.6146	0.2043 – 87784.25	0.168
High blood pressure	5.3151	0.3477 – 213.9699	0.2885
High cholesterol	0.5336	0.0229 – 7.0406	0.6498
Diabetes	0.6015	0.021 – 16.3123	0.7565
Congestive Heart Failure	1.6094	0.0082 – 267.5163	0.8556
Smoking - Never	Referent		
Smoking - Current	2.5995	0.0751 – 173.4794	0.6042
Smoking - Former	1.0864	0.1218 – 9.1872	0.9376

**Table 4:** Predictors of financial distress among breast cancer survivors from multiple regression analysis

Covariate	Beta coefficient	Confidence interval	p-value
(Intercept)	4.6323	2.39 – 6.8748	1.00E-04
Age	−0.04613	−0.073 – −0.0192	0.0011
Race - White	Referent		
Race - African American	0.4245	−0.2542 – 1.1033	0.2162
Race - Other	0.6946	−0.7433 – 2.1326	0.3384
Stage - DCIS	Referent		
Stage I	−0.4663	−1.28 – 0.3474	0.2567
Stage II	0.2067	−0.708 – 1.1215	0.6533
Stage III	−0.1379	−1.2347 – 0.9588	0.8025
Stage IV	0.1291	−1.1416 – 1.3998	0.8399
Unknown Stage	−0.6052	−1.4943 – 0.284	0.1789
Education - Less than HS	Referent		
Education - Some college	0.5293	−0.4183 – 1.4769	0.2689
Education - Associate Degree	0.8975	−0.0135 – 1.8084	0.0534
Education Bachelor’s Degree	0.0108	−0.9813 – 1.0028	0.9828
Education - Graduate Degree	0.1326	−0.7972 – 1.0623	0.7768
Income - < $20,000	Referent		
Income - $20,000 – $34,999	−1.6202	−2.6414 – −0.5991	0.0023
Income - $35,000 – $49,999	−2.2768	−3.3355 – −1.2181	1.00E-04
Income - $50,000 – $64,999	−2.6749	−3.7687 – −1.5811	0
Income - $65,000 – $79,999	−2.3341	−3.7195 – −0.9487	0.0013
Income - > $80,000	−1.801	−2.8544 – −0.7477	0.0011
Time since diagnosis	0.0174	−0.0184 – 0.0532	0.3359
Treatment - Radiation	0.4533	−0.2331 – 1.1397	0.1919
Treatment - Chemotherapy	−0.0344	−0.678 – 0.6093	0.9155
Treatment - Hormone	0.1802	−0.3688 – 0.7292	0.5146
Treatment - Targeted	0.1565	−1.049 – 1.3621	0.7963
High blood pressure	0.3042	−0.3237 – 0.9321	0.3371
High cholesterol	0.1525	−0.4805 – 0.7854	0.6322
Diabetes	0.2215	−0.4892 – 0.9322	0.5361
CHF	−0.1209	−1.4663 – 1.2245	0.8582
Smoking - Never	Referent		
Smoking - Current	0.4856	−0.4319 – 1.4031	0.2946
Smoking - Former	0.1589	−0.4274 – 0.7453	0.5902
